# Competitive sorption of Ni and Zn at the aluminum oxide/water interface: an XAFS study

**DOI:** 10.1186/s12932-018-0054-7

**Published:** 2018-03-27

**Authors:** Wenxian Gou, Matthew G. Siebecker, Zimeng Wang, Wei Li

**Affiliations:** 1 Key Laboratory of Surficial Geochemistry, Ministry of Education, School of Earth Sciences and Engineering, Nanjing University, Nanjing, 210023 China; 2 Delaware Environmental Institute, University of Delaware, Newark, DE 19716 USA; 3 Department of Civil and Environmental Engineering, Louisiana State University, Baton Rouge, LA 70803 USA

**Keywords:** Ni, Zn, Cosorption, Layered double hydroxide (LDH), XAFS

## Abstract

Trace metals (e.g. Ni, Zn) leached from industrial and agricultural processes are often simultaneously present in contaminated soils and sediments. Their mobility, bioavailability, and ecotoxicity are affected by sorption and cosorption at mineral/solution interfaces. Cosorption of trace metals has been investigated at the macroscopic level, but there is not a clear understanding of the molecular-scale cosorption processes due to lack of spectroscopic information. In this study, Ni and Zn cosorption to aluminum oxides (γ-Al_2_O_3_) in binary-sorbate systems were compared to their sorption in single-sorbate systems as a function of pH using both macroscopic batch experiments and synchrotron-based X-ray absorption fine structure spectroscopy. At pH 6.0, Ni and Zn were sorbed as inner-sphere surface complexes and competed for the limited number of reactive sites on γ-Al_2_O_3_. In binary-sorbate systems, Ni had no effect on Zn sorption, owning to its lower affinity for the metal oxide surface. In contrast, Zn had a higher affinity for the metal oxide surface and reduced Ni sorption. At pH 7.5, Ni and Zn were sorbed as mixed-metal surface precipitates, including Ni–Al layered double hydroxides (LDHs), Zn–Al LDHs, and likely Ni–Zn–Al layered triple/ternary hydroxides. Additionally, at pH 7.5, Ni and Zn do not exhibit competitive sorption effects in the binary system. Taken together, these results indicated that pH critically influenced the reaction products, and provides a crucial scientific basis to understand the potential mobility, bioavailability, and ecotoxicity of Ni and Zn in natural and contaminated geochemical environments.
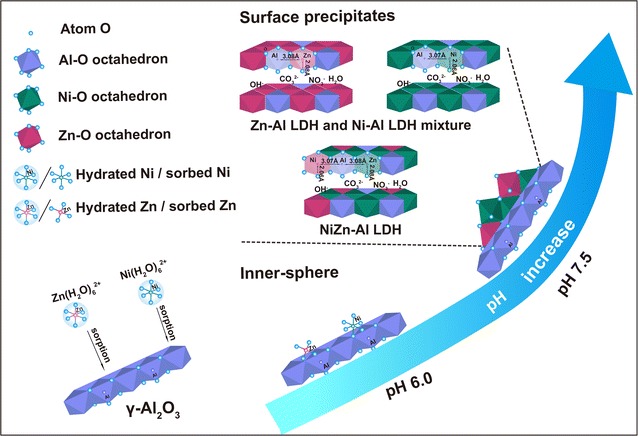

## Introduction

Trace metals discharged from anthropogenic activities such as mining, metallurgy, the burning of fossil fuels, and other urban activities pose a severe threat to soil quality and water safety, because trace metals can be present in persistent and toxic substances that bioaccumulate through the food chain and eventually influence human health [1–3]. In terrestrial ecosystems, metal sorption by soil minerals is an important interfacial process for maintaining environmental quality by removing trace metals from the solution phase. Sorption reactions also influence metal speciation in soil systems, which in turn affect metal mobility, bioavailability, and toxicity near the earth’s surface [4–6]. In addition, because the co-occurrence of multiple metals is ubiquitous in environments such as contaminated soils and sediments, the competitive effect from coexisting metals in solution for a limited quantity of mineral surface sites may dramatically intensify the potential environmental risk [7, 8]. Thus, a thorough understanding of cosorption reactions at mineral-solution interfaces can help to predict their fate in soil systems and facilitate successful environmental remediation procedures.

In the past several decades, batch method has been successfully applied to investigate metal sorption at mineral-solution interfaces. It was revealed that metal sorption is affected by environmental conditions, such as pH and temperature of solution [5, 9, 10]. Specifically, sorption of some cationic metals on oxides increases with increasing pH [5, 9], whereas metal sorption can decrease with decreasing temperature because of the endothermic energetics [10]. In addition, macroscopic sorption behaviors of single metal can be strongly affected by coexisting metal cations in solution. Murali and Aylmore studied the competition sorption behavior of several bivalent cations (e.g., Ca, Pb, Mg. Na, and Zn) [11], which displayed a decrease of Ni sorption caused by Cu competition in calcium-saturated soil system [12]. Bradbury and Baeyens also reported that Zn can suppress Ni sorption onto montmorillonite [14], whereas Flogeac et al. found that coexisting Cu and Cr could suppress Zn sorption by 62% [13]. Except for the selectivity of mineral affinity to metals, pH value has been reported as an important factor to influence competition. For example, coexisting Cu and Ni strongly competed for montmorillonite at pH < 7.0, while a much weaker competitive effect was observed at pH > 7.0 [15].

Theoretical models, such as the sorption isotherm equations, are widely used to simulate the macroscopic competition of metals [16–19]. For example, surface complexation model successfully simulates Pb and Cu sorption onto hematite over the pH range from 3 to 7 [19]. However, it is worth noting that such macroscopic data and surface complexation models cannot provide the concrete mechanistic information. In contrast, extend X-ray absorption fine structure spectroscopy (EXAFS) is a cutting-edge technique to determine atomic-scale sorption mechanisms of trace metals in single-sorbate systems, [20–24] providing local structural information (usually within 5 Å) such as coordination number, bond distances [20]. Using EXAFS, Voegelin and Kretzschmar reported that Ni and Zn could form a mixed Ni and Zn precipitates in the form of layered double hydroxide (LDH) during sorption onto soils at pH 7.4 [25]. As this experiment was conducted in complex soil system in column experiments, it is unknown whether these mixed ZnNiAl LDH formed via a co-precipitation process or a surface induced precipitation. Additionally, Ni and Zn sorption at lower pH, such as pH 6.0, was not discussed. Therefore, in this research, we aim to study the cosorption of two commonly occurring divalent trace metals contaminants (Ni and Zn) at pH 6.0 and 7.5, and use XAFS to reveal the molecular level mechanism.

## Methods

### Chemicals and reagents

The absorbent used in this study is gamma-phase aluminum oxides (γ-Al_2_O_3_), obtained from Sigma-Aldrich. The γ-Al_2_O_3_ has a strong sorption capacity for Ni and Zn [26–29], and it can also serve as an analogue to commonly found surfaces in soils and sediments such as aluminum oxides, aluminum hydroxides, and phyllosilicates (e.g., kaolinite, illite). Detailed physical and chemical properties of γ-Al_2_O_3_ can be found in a previous study [29], which reported an average particle size of 10–20 nm and a Brunauer–Emmett–Teller (BET) specific surface area of 90.1 m^2^ g^−1^. Reagents such as Zn(NO_3_)_2_·6H_2_O, Ni(NO_3_)_2_·6H_2_O, 2-morpholinoethane-sulfonic acid (MES), 2-morpholinoethane-sulfonic salt (MES-slat), 4-(2-hydroxyethyl)-1-piperazineethanesulfonic acid (HEPES), and 4-(2-hydroxyethyl)-1-piperazineethanesulfonic salt (HEPES-salt) (purity > 99%) were purchased from Sigma-Aldrich (St. Louis, MO, USA).

### Macroscopic sorption experiments

The macroscopic sorption of Ni and Zn to γ-Al_2_O_3_ was performed at ambient temperature using a batch technique. Prior to reaction, a 0.10 g dry γ-Al_2_O_3_ powder was suspended in 40 ml of 0.1 M NaNO_3_ solution for overnight for pre-equilibrium, with the suspension pH maintaining at 6.0 and 7.5 via a 50 mM MES and HEPES buffer, respectively. Previous studies indicated that MES and HEPES do not significantly interfere with Ni and Zn sorption at mineral-solution interfaces [30, 31]. Subsequently, a small amount of Ni, Zn, or mixed Ni–Zn stock solution was dispensed into the suspension to generate a desired initial metal concentration. While the metal stock solution was added to the γ-Al_2_O_3_ suspension, the suspension was rigorously stirred to prevent the formation of precipitates resulting from a possible local oversaturation. The aqueous speciation of 0.8 mM metals at pH 7.5 was calculated by Visual MINTEQ 3.1 [32]. The calculation revealed that Zn occurs dominantly as Zn(H_2_O)_6_^2+^ (89.8%) with a small contribution of ZnNO_3_(H_2_O)_5_^2+^ (8.3%), and Ni occurs dominantly as Ni(H_2_O)_6_^2+^ (91.4%) with a small contribution of NiNO_3_(H_2_O)_5_^2+^ (8.5%). Thus, the reaction conditions were unsaturated with respect to solid phase Ni(OH)_2_ and Zn(OH)_2_. After the addition of metal stock solution, the pH of the reaction suspension was adjusted immediately to a desired level (i.e., pH 6.0 or 7.5) via 0.1 M HNO_3_ or 0.1 M NaOH. After reaction for the desired time (0.5–48 h for metals sorption kinetics at pH 7.5, only 48 h for metals sorption at pH 6.0) the suspension was centrifuged for solid/solution separation. The supernatant was then passed through a mixed cellulose esters (MCE) membrane filter (0.22 μm pores) and analyzed for Ni and Zn concentrations using inductively coupled plasma-optical emission spectroscopy (ICP-OES). The amount of metal sorption was calculated through the difference between the known initial and the final aqueous concentrations. Selected fresh samples after 48 h reaction were prepared for EXAFS analysis. Sorption kinetics were fitted with first-order [33, 34] and second-order kinetic models using Excel2013 [34].

### Preparation of model compounds for EXAFS analysis

Zn–Al LDH, Ni–Al LDH, and Zn–Ni–Al LTH were prepared as model compounds via a coprecipitation method at pH 7.5 modified from Taylor [35], Sang [36], and Voegelin [25]. Briefly, a 50 ml quantity of H_2_O was first added to a reaction vessel and stirred vigorously using magnetic stirrers. The pH was adjusted to 7.5 by the addition of several drops of a mixed solution, which consisted of 1.2 M NaOH and 0.8 M Na_2_CO_3_. Mixed metal solution were prepared, containing 20 mM Al(NO_3_)_3_ together with either (1) 40 mM Ni(NO_3_)_2_ or (2) 40 mM Zn(NO_3_)_2_ or (3) 20 mM Ni(NO_3_)_2_ and 20 mM Zn(NO_3_)_2_. The mixed base solution and the mixed metal solution were added to the reaction vessel at the rates which kept the reaction pH at 7.5 ± 0.2. In this way, the reaction pH was maintained constant from the very beginning of the coprecipitation process. When the two solutions mixed with each other, a precipitate immediately formed and an opaque suspension was obtained. The suspension was further aged for 24 h at room temperature (25 °C) and centrifuged to obtain the solid. Finally, the solid was washed three times with DI water and dried in an oven for 24 h at 105 °C to obtain the final products. These final solids were verified to be a pure hydrotalcite-phase using XRD. The elemental ratios in any particle of all solids are near the initial as evidenced by TEM-EDS.

### XAFS data collection and analysis

XAFS spectra were collected for both sorption samples and model compounds at beamline 14W1 at the Shanghai Synchrotron Radiation Facility (SSRF) and at beamline 1W2B at the Beijing Synchrotron Radiation Facility (BSRF). The electron storage ring at SSRF operated at 3.5 GeV and with an average current of 300 mA, and the electron storage ring operated at 2.5 GeV and with an average current of 250 mA at BSRF, respectively. In both beamlines, a pair of Si(111) monochromator crystals were employed, which were detuned by 50% to suppress high order harmonic X-rays. For Ni and Zn K-edge XAFS data collection, Ni and Zn foil were used for energy calibration, respectively.

Ni/Zn-reacted wet paste samples were packed in plastic sample holders, which were sealed with thick Kapton tape. The sample holders were then placed at a 45° angle to the incident X-ray beam. The spectra were collected in fluorescence mode using a Lytle detector, which was filled with pure Ar_2_ and positioned at a 90° angle to the incident beam. For model compounds, dry powders were ground to a particle size of < 38 μm using an agate mortar and then adhered evenly to Kapton tape [37]. The tape was folded multiple times to ensure homogeneity and uniformity of the sample in the X-ray beam. EXAFS data for these samples were collected in transition mode, where gas ionization chambers were employed and filled with pure nitrogen or a mixture of nitrogen and argon to obtain 15–25% X-ray beam absorption rate for I_0_ and near 75–85% for I_1_, respectively. For sorption samples prepared at pH 6.0, only X-ray absorption near-edge structure (XANES) spectra of Ni- and Zn-reacted samples were collected because of the low signal-to-noise ratio. The XANES spectra of Ni + Zn Alox (Alox refers to Al oxide), Zn Alox, and Zn + Ni Alox were collected in fluorescence mode using Lytle detector, whereas the XANES of Ni Alox at pH 6.0 was collected in fluorescence mode using a 13-units multi-element Germanium Detector. For all samples, multiple scans were taken to obtain decent signal to noise ratio.

The EXAFS data analysis was accomplished by IFEFFIT program package [38]. Raw spectra were averaged and background subtracted with a spline function to obtain the χ(*k*) function. The χ(*k*) function with *k*^3^-weighting was then Fourier transformed. Shell-by-shell fitting was done in R-space. Theoretical scattering paths were calculated based on the crystal information file of Nikischerite [39] from the American Mineralogy Crystal Structure Database. Nikischerite is a LDH type mineral, which consists of planar sheets of octahedral [AlFe^2+^(OH)_6_] with octahedral [Na(H_2_O)_6_], and tetrahedral (SO_4_) and H_2_O in the interlayer [39]. We replaced Fe with Ni or Zn in the Nikischerite structure to calculate the theoretical scattering paths for Ni–O, Ni–Ni and Ni–Al or the corresponding Zn–O (Zn/Al) paths. The σ^2^ values, which describe the thermal and static disorder, were linked together for shells of similar distance to the center atom (Ni–Al and Ni–Ni/Zn for Ni-rich sample and model compounds, and Zn–Al and Zn–Zn/Ni for Zn-rich sample and model compounds) for second shell fitting. The amplitude reduction factors (*S*_*0*_^*2*^) were estimated to be 0.93 and 0.85 based on the fitting of Ni(NO_3_)_2_ solution and Zn(NO_3_)_2_ solution. In case of Ni–Zn–Al LTH, because Zn and Ni are neighboring elements in the periodic table, their backscattering is similar and hard to distinguish from each other as second-shell backscatters. Thus, the back scattering paths of second-shell are shown as Ni–Ni/Zn or Zn–Zn/Ni. Estimated errors for the first shell are ± 20% for coordination number, ± 0.01 Å for bond distance and ± 0.001 Å^2^ for Debye–Waller factors, and for second shell ± 40% for coordination numbers, ± 0.04 Å for bond distances and ± 0.005 Å^2^ for Debye–Waller factors.

## Results and discussion

### Macroscopic sorption of Ni and Zn on Al oxide

#### Sorption kinetics of Ni and Zn in single-sorbate systems at pH 7.5

**Fig. 1:**
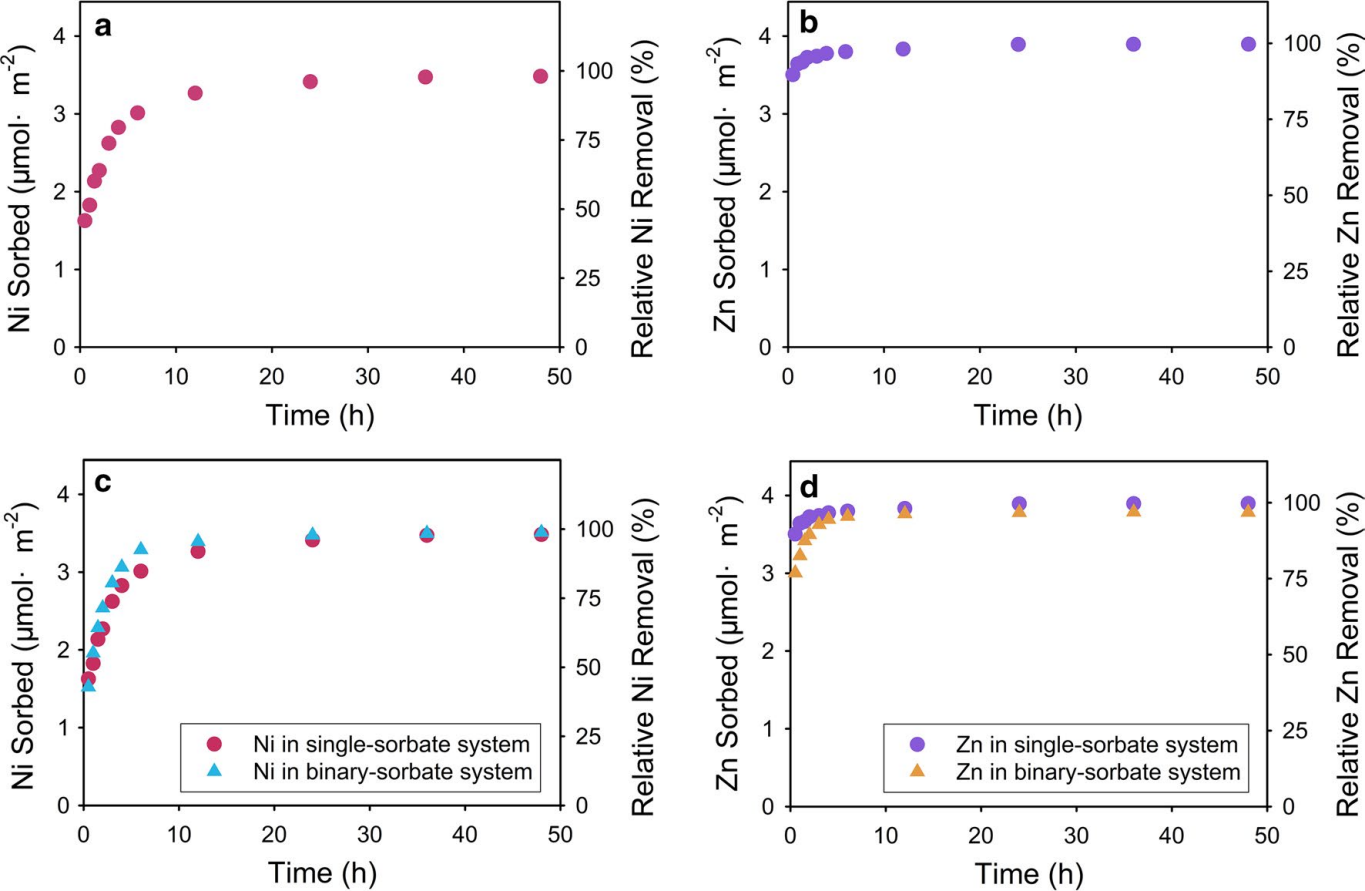
Kinetics of Ni and Zn sorption onto γ-Al_2_O_3_ from 0.8 mM Ni or Zn solutions at pH 7.5. **a** Ni sorption onto γ-Al_2_O_3_ in the single-sorbate system; **b** Zn sorption onto γ-Al_2_O_3_ in the single-sorbate system. **c** Ni sorption onto γ-Al_2_O_3_ in the binary-sorbate system; **d** Zn sorption onto γ-Al_2_O_3_ in the binary-sorbate system. A constant ionic strength of 0.1 M NaNO_3_ and a solid/solution ratio of 2.5 g l^−1^ was used. The last sample of each experiment was collected and analyzed by EXAFS

Figure 1 shows the kinetics of Ni and Zn sorption to γ-Al_2_O_3_ at pH 7.5 in single-sorbate systems. The trends of sorption kinetics are well in line with the typical trends seen for trace metals [40–43]. Specifically, metal sorption is initially fast and occurs on a time scale of minutes to hours and approximately 84.8% (3.0 μmol m^−2^) of the initial Ni and 98.9% (3.8 μmol m^−2^) of the initial Zn are sorbed within the first 6 h. After a reaction time of 48 h, Ni sorption and Zn sorption are nearly complete. Roberts et al. reported that 75% of initial Ni is sorbed onto soil clay from a 3 mM solution at pH 7.5 within in the first 12 h, and Ni sorption is nearly complete after 200 h [40]. Roberts et al. reported that 80% of initial Zn is sorbed to silica surface at pH 7.5 within 15 min, and 100% Zn removal is achieved within 3 h. In a high surface area gibbsite system, 80% of initial Zn is removed after 24 h and nearly complete Zn sorption is achieved after 200 h. In a low surface area gibbsite system, only 50% of initial Zn is removed [41]. The initial rapid stage is explained as adsorption to surface sites with high reactivity, and the later slow stage is attributed to precipitation, diffusion into the mineral lattice, or adsorption to sites with lower reactivity [40].

#### Cosorption of Ni and Zn in binary-sorbate systems at pH 7.5

Figure 1a compares the kinetics of Ni and Zn sorption in a binary-sorbate system with that in a single-sorbate system at pH 7.5. Similar to the sorption kinetics in the single-sorbate system, Ni and Zn sorption continuously increase with time in the binary-sorbate system. There is also a similar two-phase sorption trend, with an initially rapid sorption period followed by a slower sorption period. Ni and Zn sorption in the binary-sorbate system overlap almost completely with that in the single-sorbate system, suggesting that the coexisting aqueous Zn and Ni cations have no measurable effect on Ni or Zn sorption in binary-sorbate systems at pH 7.5. After a reaction time of 6 h, 92.6% (3.3 μmol m^−2^) of initial Ni and 97.2% (3.73 μmol m^−2^) of initial Zn are sorbed; and after 48 h, Ni sorption and Zn sorption achieve nearly complete. In other words, Ni and Zn did not exhibit a remarkable competitive sorption effect on each other in binary-sorbate systems at pH 7.5.

The sorption kinetics of Ni and Zn sorption at pH 7.5 were quantitatively modeled via both pseudo first-order [33, 34] and second-order kinetic models [34]. The pseudo first-order kinetic model is expressed in Eq. (1):

1 $$\ln (q_{e} - q_{t} ) = \ln q_{e} - k_{1} t$$

where q_e_ (mg g^−1^) and q_t_ (mg g^−1^) stand for the amount of metal ions sorption onto the surface of Al oxides at equilibrium condition and at a given reaction time t (h), k_1_ (mg g^−1^ h^−1^) refers to a rate constant of metal ions sorption onto the surface of the Al oxide in a pseudo first-order kinetic model. To fit Eq. (1) to experimental data, the value of q_e_ was approximated to the maximal sorption, and k_1_ were determined by the slope of plots. When k_1_ has been obtained, q_e_ were calculated from the intercepts and tabulated in Table 1. In addition, because pseudo first-order kinetic model is usually applicable over the initial reaction time of the adsorption process, the fitting process was operated only for the initial 6 h.

**Table 1:** Parameters for the Ni and Zn sorption kinetics on γ-Al_2_O_3_ in single- and binary-sorbate systems at pH 7.5

Sample	First second order			Pseudo second order		
	q_e_ (mg^−1^ g^−1^)	k_1_ (mg^−1^ g^−1^ h^−1^)	R^2^	q_e_ (mg^−1^ g^−1^)	k_2_ (mg^−1^ g^−1^ h^−1^)	R^2^
Ni in single-sorbate system	11.87	0.396	0.995	20.00	0.05	0.998
Ni in binary-sorbate system	13.21	0.258	0.981	22.22	0.06	0.994
Zn in single-sorbate system	4.81	0.230	0.880	22.73	0.97	1.000
Zn in binary-sorbate system	1.95	0.471	0.961	22.73	0.28	1.000

**Fig. 2:**
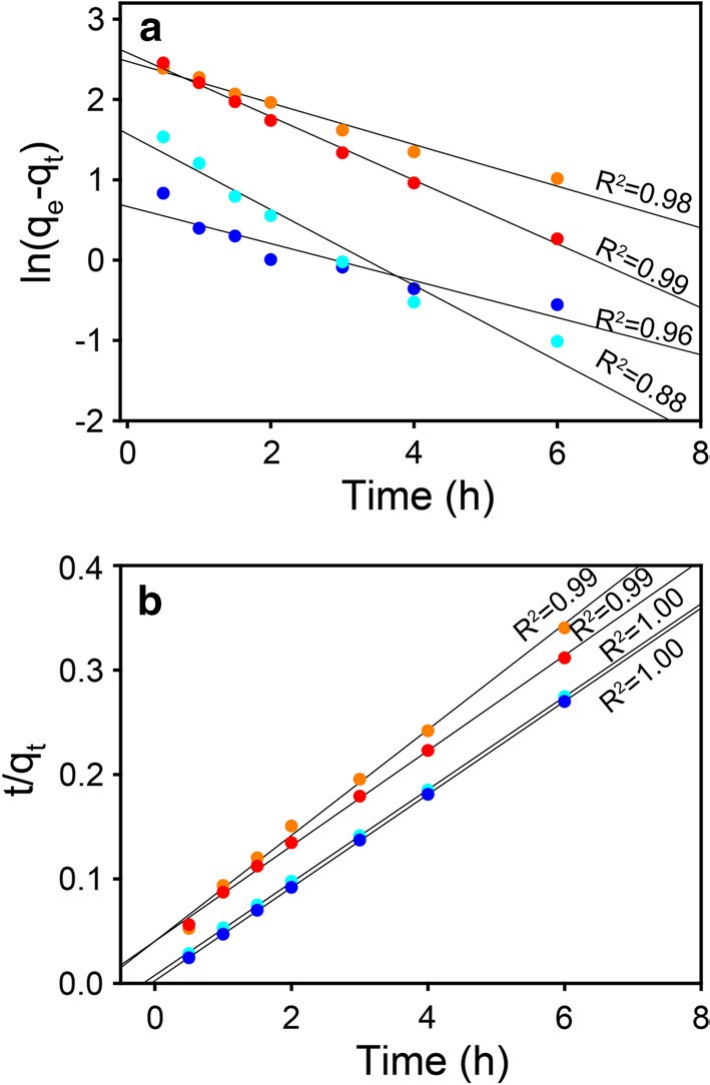
Pseudo first-order (**a**) and pseudo second-order (**b**) kinetic plots of Ni and Zn sorption on γ-Al_2_O_3_ from 0.8 mM initial metal concentration solution at pH 7.5. The orange circles and red circles denote Ni sorption from binary- and single-sorbate systems, respectively. The indigo circles and blue circles denote Zn sorption from binary- and single-sorbate systems, respectively. The kinetic sorption data used for models fitting were collected up to 6 h, before which the equilibrium was not reached

The pseudo second-order kinetic model is expressed in Eq. (2):

2 $$\frac{t}{{q_{t} }} = \frac{1}{{k_{2} q_{e}^{2} }} + \frac{t}{{q_{e} }}$$

where k_2_ (mg g^−1^ h^−1^) signifies a rate constant of metal ion sorption in pseudo second-order kinetic model and the other parameters is the same with Eq. (1). The values of k_2_ and q_e_ were calculated from the intercepts and slopes of the plots in Fig. 2b.

The fitting results using both kinetic models are summarized in Table 1. The correlation coefficient (R^2^) of all kinetics from pseudo first-order model (0.880–0.995) are lower than that from pseudo second-order model (0.994–1.000); the calculated q_e_ values of metals from pseudo first-order model (in case of Ni, 20.0 and 22.2 mg^−1^ g^−1^ in single- and binary-sorbate system; in case of Zn, 22.7 mg^−1^ g^−1^ in both single- and binary-sorbate system) are also closer to the measured values (in case of Ni, 20.4 and 20.6 mg^−1^ g^−1^ in single- and binary-sorbate system; in case of Zn, 22.2 and 22.8 mg^−1^ g^−1^ in single- and binary-sorbate system). The higher R^2^ values and more reasonable calculated q_e_ values suggest that the sorption process follows pseudo second-order kinetic model rather than pseudo first-order kinetic model. This result is excellent agreement with that summarized by Plazinski et al. [34], who suggested that metal sorption kinetics usually obey the pseudo second-order kinetic model instead of the pseudo first-order kinetic model. The more suitable pseudo second-order kinetic model for the two metals indicate that the rate of Ni and Zn sorption at pH 7.5 may be controlled by the adsorption/desorption process [34]. The k_2_ value from the pseudo second-order kinetic model for Ni sorption are 0.05 and 0.06 mg^−1^ g^−1^ h^−1^ in single- and binary-sorbate system, which is lower than that for Zn sorption (0.97 and 0.29 mg^−1^ g^−1^ h^−1^ in single- and binary-sorbate system). In other words, the sorption rate of Zn is faster than that of Ni, indicating that Zn may have a higher affinity than Ni for sorption to γ-Al_2_O_3_.

#### Cosorption of Ni and Zn at pH 6.0

**Fig. 3:**
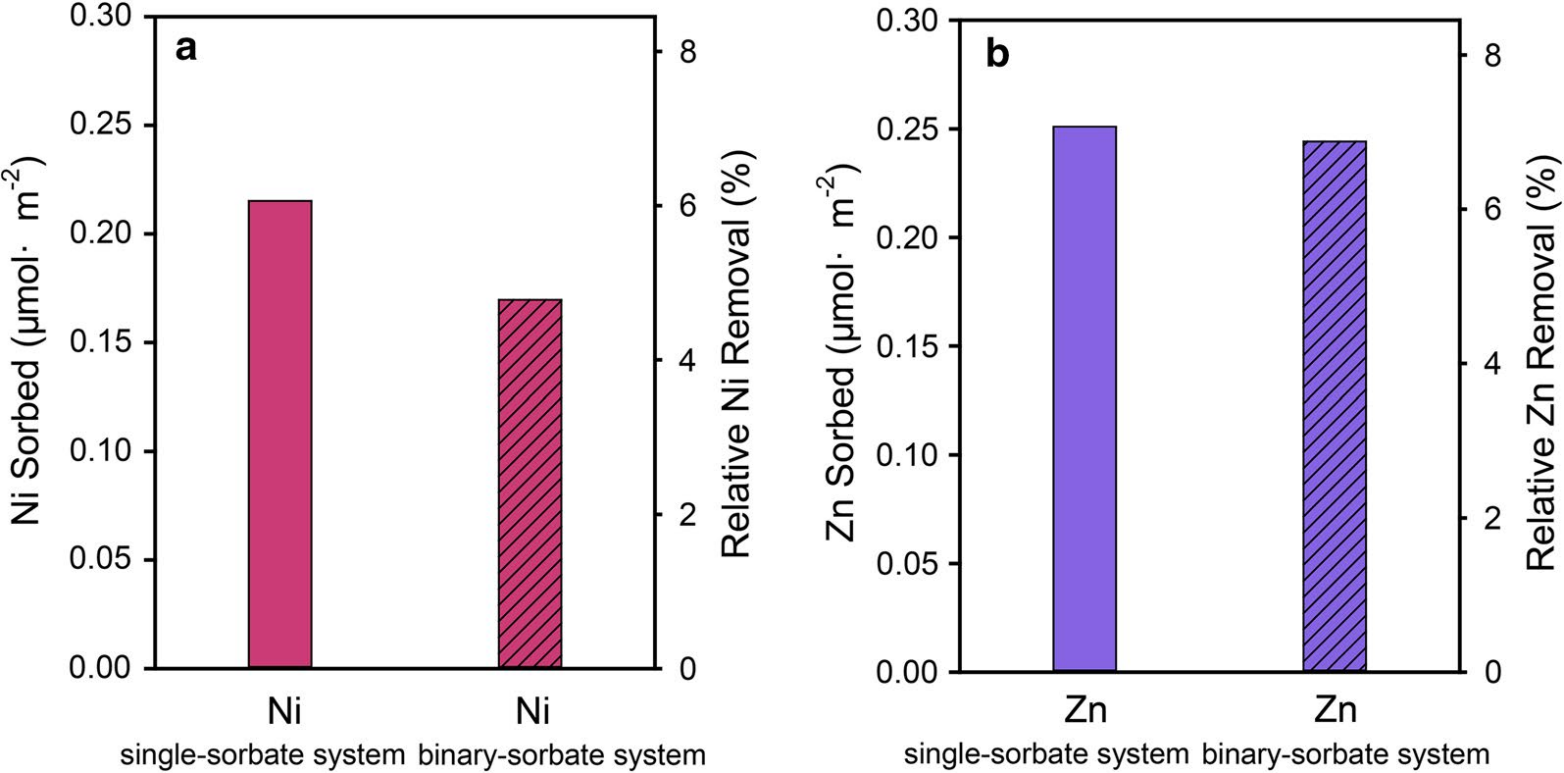
Ni (**a**) and Zn (**b**) sorption on γ-Al_2_O_3_ from 0.8 mM initial metal concentration solution at pH 6 and a constant ionic strength 0.1 M (NaNO_3_) over entire reaction period (48 h) in single- and binary-sorbate systems

At pH 6.0 only one sample reacting for 48 h was collected (due to the low amount of metal sorption) to compare sorption behaviors (Fig. 3). Approximately 6.4% of the initial Zn removal from solution in single-sorbate system, resulting in a surface density of 0.25 μmol m^−2^; in contrast, 6.2% (0.24 μmol m^−2^) of the initial Zn removal in binary-sorbate system. This means no difference is found for Zn sorption between the single- and binary-sorbate systems, which suggests that coexisting aqueous Ni cations have no measureable effect on Zn sorption. Differently, the presence of Zn reduces Ni sorption from a surface density of 0.22 μmol m^−2^ in single system to 0.17 μmol m^−2^ in the binary-sorbate systems, indicating that coexisting aqueous Zn cations can have an inhibitory effect on Ni sorption by 22.7%.

### EXAFS analysis of Ni and Zn sorption at pH 7.5

**Fig. 4:**
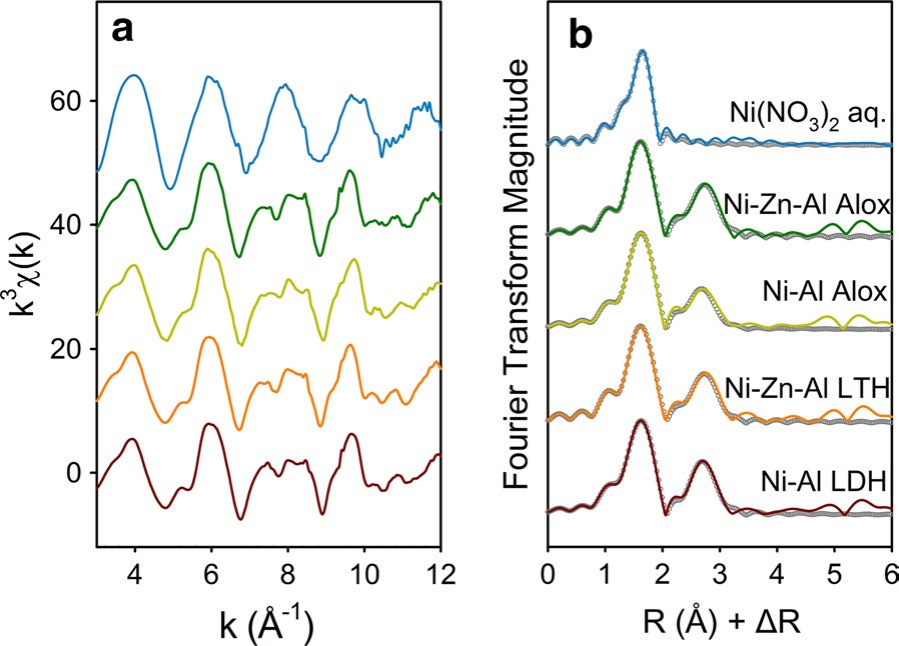
Ni K-edge EXAFS spectra of Ni-reference compounds and Ni-reacted γ-Al_2_O_3_ samples after reaction of 48 h at pH 7.5: **a** *k*^3^ weighted functions; **b** the corresponding Fourier transform uncorrected for phase shift. Experimental data are shown as colored solid lines and fitted data as dark gray circles

Figure 4 shows the Ni *k*^3^-weighted χ(*k*) EXAFS spectra and the corresponding raw and fitting Fourier transformed spectra (uncorrected for phase shift) for Ni-reacted samples at pH 7.5 and model compounds (e.g., aqueous Ni^2+^ in solution, Ni–Al LDH, and Ni–Zn–Al LTH). The *k*^3^-weighted χ(*k*) spectra reveals a truncated oscillation at about *k* of 8 Å^−1^ (Fig. 4a). This is referred to “beat pattern”, a finger-printing feature for Ni–Al LDH [44], resulting from complex interference scattering between single scattering paths of first shell O atoms and second shell metal (Me) and Al atoms in a series of multiple scattering paths [44]. The Fourier transformed (FT) of EXAFS spectra of Ni-reacted samples are similar with Ni–Al LDH and Ni–Zn–Al LTH standards (Fig. 4b), all of which exhibit two distinct scattering shells. The first peak in the FT can be fitted well at 2.06 Å with a Ni–O coordination number of 6; a similar radial distance is found for first shell O of Ni solution, Ni–Al LDH and Ni–Zn–Al LTH. The second shell is fitted to be ~ 4 Ni and ~ 2 Al with interatomic distances of 3.07 and 3.06 Å, respectively. These fitting parameters (Table 2 ) confirm the formation of Ni–Al LDH or LTH precipitates.

**Table 2:** EXAFS fitting results of Ni for model compounds and sorption samples (S_0_^2^ = 0.93)

Sample	Shells	CN	R (Å)	σ^2^ (Å^2^)	R_f_	ΔE_0_ (ev)
Ni(NO_3_)_2_ aq.	Ni–O	6.0^a^	2.06	0.005	0.0010	− 0.90
Ni–Al LDH	Ni–O	5.8	2.06	0.006		2.00
	Ni–Ni	3.5	3.07	0.007	0.0005	
	Ni–Al	0.8	3.06	0.007		
Ni–Zn–Al LTH	Ni–O	6.3	2.06	0.007		2.86
	Ni–Ni/Zn	3.5	3.07	0.008	0.0010	
	Ni–Al	1.3	3.06	0.008		
Ni–Al Alox	Ni–O	5.3	2.06	0.005		2.56
	Ni–Ni	3.4	3.06	0.008	0.0014	
	Ni–Al	1.9	3.05	0.008		
Ni–Zn–Al Alox	Ni–O	5.5	2.06	0.005		2.57
	Ni–Ni/Zn	4.1	3.07	0.008	0.0010	
	Ni–Al	1.5	3.06	0.008		

Estimated errors for the first shell are ± 20% for coordination number, ± 0.01 Å for bond distance and ± 0.001 Å^2^ for Debye–Waller factors, and for second shell ± 40% for coordination numbers, ± 0.04 Å for bond distances and ± 0.005 Å^2^ for Debye–Waller factors

*CN* coordination number and were determined by the fit, *R* the interatomic distance in Å, *σ*^*2*^ the Debye–Waller factor in Å^2^, *R*_*f*_ the absolute misfit between data and theory, *ΔE*_*0*_ the energy shift in eV

^a^Fixed value

**Fig. 5:**
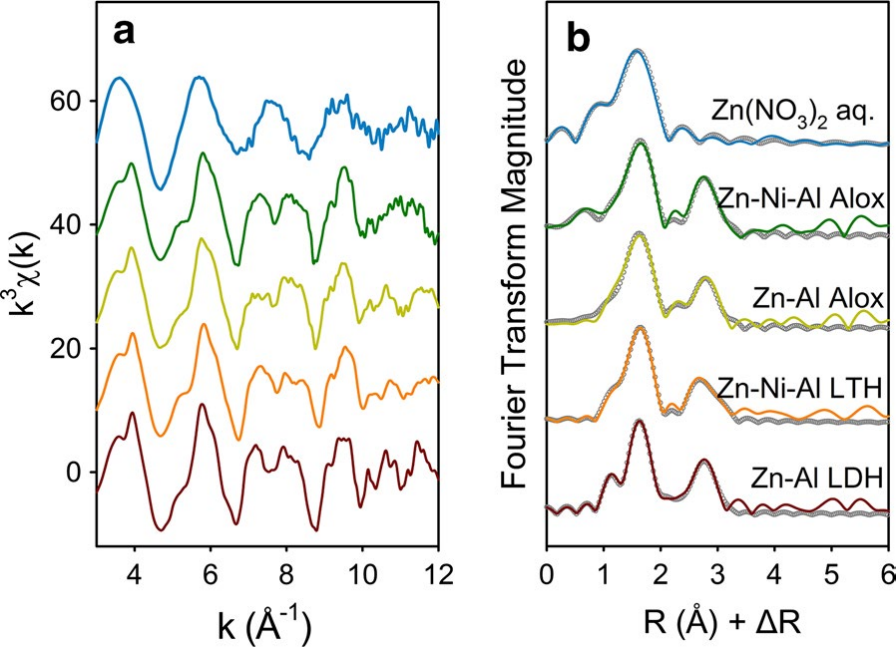
Zn K-edge EXAFS spectra of Zn-reference compounds and Zn-reacted γ-Al_2_O_3_ samples after reaction of 48 h at pH 7.5: **a** *k*^3^-weighted functions; **b** the corresponding Fourier transform uncorrected for phase shift. Experimental data are shown as colored solid lines and fitted data as dark gray circles. Ni–Zn–Al LDH (in Fig. 4) and Zn–Ni–Al LDH (in this figure) are the same sample and used to refer the spectrum collected from Ni and Zn K-edges, respectively. Ni–Zn–Al Alox (Fig. 4) and Zn–Ni–Al Alox (in this figure) are the same sample and used to refer the spectrum collected from Ni and Zn K-edges, respectively

Similar to the Ni-reacted samples, the features in the Zn K-edge EXAFS spectra for Zn-reacted samples at pH 7.5 are similar with each other and consistent with the Zn–Al LDH and Zn–Ni–Al LTH (Fig. 5). Two obvious peaks (Fig. 5b) occur at ~ 1.6 and ~ 2.4 Å and result from the backscattering of first shell O atoms and second shell Me (Zn, Ni, and Al) atoms, respectively. In addition, a similar truncated oscillation also is observed at *k* of ~ 8 Å^−1^ in *k*^3^-weighted spectra (Fig. 5a). Fitting results (Table 3) indicate that precipitated Zn is present in an octahedral environment with 6 O atoms at an interatomic distance of 2.08 Å in the first shell. About 2 Al atoms at 3.09 Å and 4 Zn atoms at 3.08 Å are present in the second shell. These parameters are consistent with those for Zn–Al LDH and Ni–Zn–Al LTH model compounds.

**Table 3:** EXAFS fitting results of Zn for model compounds and sorption samples (S_0_^2^ = 0.85)

Sample	Shells	CN	R (Å)	σ^2^ (Å^2^)	R_f_	ΔE_0_ (ev)
Zn(NO_3_)_2_ aq.	Zn–O	6.0^a^	2.06	0.006	0.0002	1.16
Zn–Al LDH	Zn–O	6.5	2.08	0.008		0.89
	Zn–Zn	3.5	3.10	0.010	0.0005	
	Zn–Al	2.4	3.09	0.010		
Zn-Ni–Al LTH	Zn–O	6.6	2.08	0.008		2.85
	Zn–Zn/Ni	4.6	3.08	0.010	0.0010	
	Zn–Al	1.6	3.07	0.010		
Zn–Al Alox	Zn–O	6.2	2.06	0.009		1.23
	Zn–Zn	4.3	3.09	0.010	0.0015	
	Zn–Al	2.4	3.08	0.010		
Zn–Ni–Al Alox	Zn–O	6.9	2.08	0.010		1.182
	Zn–Zn/Ni	5.3	3.09	0.011	0.0018	
	Zn–Al	1.4	3.08	0.011		

Estimated errors for the first shell are ± 20% for coordination number, ± 0.01 Å for bond distance and ± 0.001 Å^2^ for Debye–Waller factors, and for second shell ± 40% for coordination numbers, ± 0.04 Å for bond distances and ± 0.005 Å^2^ for Debye–Waller factors

*CN* coordination number and were determined by the fit, *R* the interatomic distance in Å, *σ*^*2*^ the Debye–Waller factor in Å^2^, *R*_*f*_ the absolute misfit between data and theory, *ΔE*_*0*_ the energy shift in eV

^a^Fixed value

Examination of Ni and Zn K-edge EXAFS spectra suggests that Ni and Zn sorption to Al oxide at pH 7.5 involves formation of surface precipitates, as strictly adsorbed metals and aqueous Zn and Ni cations (i.e., formation of inner-sphere surface complexes) do not have the significant scattering from second coordination shells seen in the FT of our samples. The formation of precipitates is probably because high concentration of Ni or Zn is used in this study. Previous studies for both Zn and Ni adsorbed to other Al oxides indicated surface precipitation as LDH phases favored at higher metal concentration (e.g. > 0.4 mM for Zn) or high sorption density (e.g. 1.5 μmol m^−2^) [26, 45]. In comparing the EXAFS spectra for metals sorption in binary-sorbate system to that in single-sorbate system, the similarities between the two systems indicate no obvious effect on the sorption mechanism in the binary-sorbate system at pH 7.5.

### XANES analyses for Ni and Zn sorption at pH 6.0

**Fig. 6:**
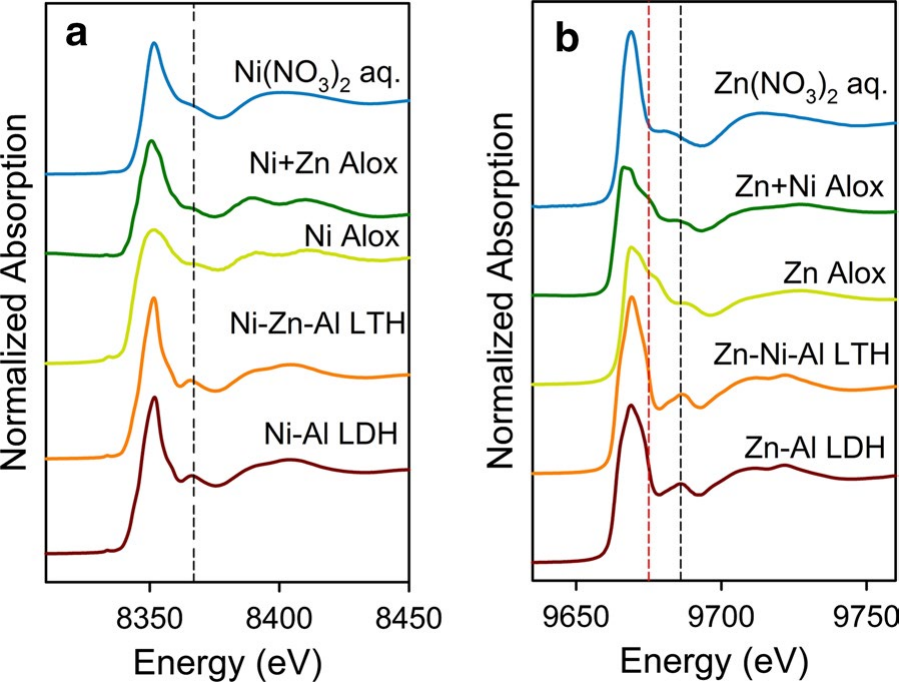
Ni K-edge XANES spectra of reacted samples after reaction of 48 h at pH 6.0 (**a**) and Zn K-edge XANES spectra of reacted samples at pH 6.0 (**b**). The spectra of Ni–Al LDH, Ni–Zn–Al LTH, Zn–Al LDH and Zn–Ni–Al LTH are shown for reference

All normalized Ni and Zn K-edge XANES spectra of the sorption samples and model compounds are presented in Fig. 6. Figure 6a compares the normalized Ni XANES spectra of the sorption sample (Ni Alox and Ni + Zn Alox) to those of Ni–Al LDH, Ni–Zn–Al LTH, and the aqueous Ni in solution. This comparison revealed that surface precipitates as Ni-rich LDH are not a dominant species during Ni sorption at pH 6.0 in the single sorbent system. A distinguishable shoulder feature at ~ 8367 eV occurs in the spectra (dashed black line) for the solid phase precipitates Ni–Al LDH and Ni–Zn–Al LTH but is less pronounced in Ni sorption samples or Ni solution. This shoulder feature is interpreted as the multiple scattering of the photoelectron between the central atom (Ni) and its neighboring atoms (e.g., O, Al, Ni/Zn) [46, 47]. In addition, the oscillation over the range of 8375–8435 eV in the Alox sorption samples is different from the broad peak for Ni in solution. There is a distinct split in this oscillation at about 8400 eV. This indicates that the mechanism of Ni sorption at pH 6.0 neither out-sphere surface complexion nor coprecipitation but rather inner-sphere surface complexion. This split is more pronounced in the binary Ni + Zn Alox sorption sample than in the single metal Ni Alox sorption sample and the LDH/LTH standards. The large split in Ni + Zn Alox at about 8400 eV indicates Ni is bound either as an inner-sphere surface complex or completely incorporated into the octahedral layer of a gibbsite-like sheet. Either way there is a low amount of transition metal as second nearest neighbors, indicating that no LDH/LTH surface precipitate has formed. This split commonly occurs when a heavier transition metal is surrounded by a lighter element, such as Al, in an octahedral sheet [45, 48–51]. However, as heavier atoms, such as Ni and Zn, begin to populate the second shell (as would occur in an LDH or LTH type phase), the split begins to disappear [48–50, 52].

Figure 6b shows the normalized Zn K-edge XANES of sorption samples at pH 6.0 along with the Zn–Al LDH, Ni–Zn–Al LTH, and aqueous Zn cation standards. Different features are observed between the sorption samples and model compounds. For example, the deficiency of the peak intensity at ~ 9686 eV in the sorption samples (dashed black line) versus the solid phase LDH/LTH precipitates implies changes of Zn coordination from octahedral to tetrahedral [53], and suggested that the sorption mechanisms may not be surface precipitation. In addition, there is a distinct shoulder at ~ 9675 eV (dashed red line), which is absent in the spectrum of Zn–Al LDH, Ni–Zn–Al LTH, and aqueous Zn solution. Such a shoulder is seen in previous studies, which suggests the central Zn atom is present in tetrahedral environment, surrounded by four oxygen atoms [27, 54–56], corresponding to a typical inner-sphere complexes. The differences between the spectra of the Alox sorption samples and the LDH/LTH precipitates indicate that at pH 6 the principle sorption species are not surface precipitates. Thus, adsorption as inner-sphere surface complexes would to the best extent explain the main mechanism Ni and Zn retention to γ-Al_2_O_3_ at pH 6.0.

### Impacts of the binary versus single sorbate systems

**Fig. 7:**
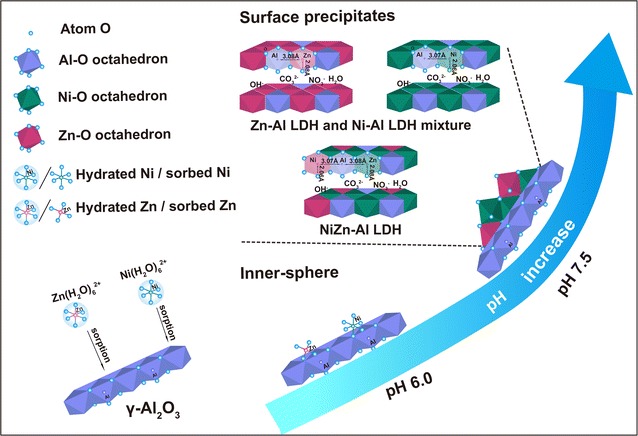
Schematic illustration of the cosorption of Zn and Ni at γ-alumina/water interface

This study shows that the behaviors and sorption mechanisms (i.e., adsorption versus precipitation) of Ni and Zn to γ-Al_2_O_3_ are largely affected by pH values (Fig. 7). At pH 6, inner-sphere adsorption of Ni and Al appears to dominate, and as pH increases the major species become LDH and LTH precipitates. At the end of the reaction at pH 6.0, the macroscopic data indicate Zn is sorbed with no obvious interference from coexisting aqueous Ni cations, whereas the amount of Ni sorption is slightly reduced by 22.7% of the total Ni by coexisting aqueous Zn (Fig. 3b). Ni and Zn K-edge XANES data indicate they are sorbed to γ-Al_2_O_3_ as inner-sphere surface complexes; however, inner-sphere adsorption can strongly depend on mineral surface reactive sites [7]. Because Ni and Zn are neighboring elements in the periodic table and have similar chemical properties (e.g., both exhibit a +2 valence state, which is stable in many natural geochemical environments, and both can form LDH precipitates), they are reasonably expected to compete for the limited number of surface reactive sites on γ-Al_2_O_3_. This competition should result in a decrease of their sorption amounts in the binary-sorbate system versus the single-sorbate system [12, 57, 58].

However, the slightly higher amount of Zn sorption than Ni (Fig. 3a, b) suggests that Zn may have a marginally higher affinity for sorption to γ-Al_2_O_3_. This finding is supported by the slight decrease in the amount of Ni bound to γ-Al_2_O_3_ in the binary-sorbate system and the slightly higher k_2_ values during Zn sorbed onto γ-Al_2_O_3_ than during Ni sorbed both in single- and binary-sorbate system. This selectivity sequence has been observed during Zn (pH_50_ = 5.4) and Ni (pH_50_ = 6.3) sorption in Al gel systems [59], where value of pH_50_ is the pH at which 50% adsorption occurs [60]. A possible explanation is the difference in their first hydrolysis constants. Theoretically, the metal with a lower first hydrolysis constant is expected to possess a higher affinity for similar sites on a mineral surface [61]. Thus, the slightly lower first hydrolysis constant of Zn (9.0) than Ni (9.6) is responsible for its slightly higher competitive behavior for the γ-Al_2_O_3_ surface [62].

At pH 7.5, Ni and Zn do not compete substantially with each other in the binary-sorbate system. At equilibrium, there are essentially the same amounts of Ni and Zn sorbed to γ-Al_2_O_3_ in both the binary and single sorbate systems. Although, the initial Ni and Zn sorption rates are slightly increased and decreased in the binary systems, respectively (Fig. 1). The EXAFS results indicate that Ni and Zn are sorbed on γ-Al_2_O_3_ as surface precipitates in both sorbate systems. Surface precipitation is independent of surface sorption sites [9] while surface complexion is not. Consequently, Ni and Zn do not need to compete for the limited number of reactive sites and do not exhibit a competitive effect during sorption to γ-Al_2_O_3_ at pH 7.5.

A limitation of EXAFS is that it cannot distinguish between the simultaneous presence of the double metal LDH (e.g., Ni–Al LDH, Zn–Al LDH) and multi-metal LTH (i.e., Ni-Zn-Al LTH) phases if both were the same sample. This is because (1) the Ni and Zn are close to each other in the periodic table, and (2) both compounds would be absorbing X-rays from the same incident beam. Thus, it is challenging to conclude solely from FT fits whether (1) Ni–Zn–Al LTH has formed, or (2) pure-phases of Ni–Al LDH and Zn–Al LDH have formed independent, or (3) both LDH and LTH have formed in the binary-sorbate system. However, the following must be considered: (1) all three aqueous metal (Ni, Zn, and Al) cations are present in the solution adjacent to the γ-Al_2_O_3_ surface; (2) the solid phase (γ-Al_2_O_3_) can act as a nucleation catalyst for surface precipitation and also as a source for metal (Al^3+^) cations [52]; and (3) Zn reacts with γ-Al_2_O_3_ readily to form LDHs [26]. Lastly, above pH 4.5, dissolved Al concentrations rapidly reach saturation with respect to the solubility product constant of gibbsite [63], which is an Al hydroxide mineral composed of layered octahedral sheets. Given these environmental and our experimental conditions, a layered hydroxide (gibbsite-like phase) will form rapidly in our system and there is a high potential for the rapid incorporation of Ni, Zn, and Al to form an LTH.

In addition to the favorable aquatic chemical conditions for the formation of an LTH adjacent to the γ-Al_2_O_3_ surface, the similarities between the EXAFS spectra and fitting of the model compounds and reacted samples indicate that at the end of our sorption reactions the structures of the products are related to those of our model compounds. Also, multi-metal-rich layered hydroxides containing both Ni and Zn have been successfully synthesized by mixing Zn and Ni salts at ambient temperatures and neutral pH; this multi-metal-rich layered hydroxide is a proven product during sorption to clays in column experiments [25].

## Conclusions

In this research, we applied both macroscopic batch method and microscopic EXAFS to investigate the cosorption of Ni and Zn onto aluminum oxide. At pH 7.5, Ni and Zn do not exhibit a measurable competitive effect, whereas competitive sorption between Ni and Zn was observed at pH 6.0. This pH dependent phenomenon was explained by their different sorption mechanisms revealed by EXAFS analysis that both metals adsorbed inner-sphere surface complexes at pH 6.0 while forming LDH-type surface precipitates at pH 7.5. Based on these results, we suggested that the competitive effects between Ni and Zn on Al oxide surfaces should be considered in acidic environments, which is less significant neural or alkaline environments. The findings presented here are important for risk assessments of the toxic metal pollution, metal speciation, and surface complexation modeling. In addition, further work (e.g. transmission electron microscope, pair distribution function) will be used to look for the simultaneous presence of Ni–Zn–Al in the hydroxide sheet and their reactivity.

## Abbreviations

XAFS: X-ray absorption fine structure
EXAFS: extend X-ray absorption fine structure
XANES: X-ray absorption near-edge structure
LDH: layered double hydroxide
LTH: layered triple (or ternary) hydroxide
MES: 2-morpholinoethane-sulfonic acid
MES-salt: 2-morpholinoethane-sulfonic salt
HEPES: 4-(2-hydroxyethyl)-1-piperazineethanesulfonic salt
HEPES-salt: 4-(2-hydroxyethyl)-1-piperazineethanesulfonic salt
Alox: Al oxide
